# Heterogeneity evaluation of multi-high *b*-value apparent diffusion coefficient on cerebral ischemia in MCAO rat

**DOI:** 10.3389/fnins.2022.1048429

**Published:** 2022-12-20

**Authors:** Liwei Shi, Bo Yu, Qiuyan Chen, Tianxiu Zheng, Peiqiu Xing, Dingtai Wei

**Affiliations:** ^1^The Third Clinical Medical College, Fujian Medical University, Fuzhou, Fujian, China; ^2^Department of Radiology, Ningde Municipal Hospital of Ningde Normal University, Ningde, Fujian, China; ^3^Functional and Molecular Imaging Laboratory for Cerebral Vascular Diseases, Ningde Municipal Hospital of Ningde Normal University, Ningde, Fujian, China

**Keywords:** diffusion, ischemia, Aquaporin 4, MRI, rat

## Abstract

**Purpose:**

To assess brain damage in a rat model of cerebral ischemia based on apparent diffusion coefficient (ADC) data obtained from multi-high *b*-values and evaluate the relationship between Aquaporin 4 (AQP4) expression and ADC.

**Methods:**

Thirty eight male Sprague–Dawley rats were randomized into two groups: (1) sham controls (*n* = 6) and (2) cerebral ischemia (successful model, *n* = 19). All rats underwent diffusion-weighted imaging (DWI) with both standard *b*-values and multi-high *b*-values (2,500–4,500 s/mm^2^) using a 3.0-T device. Standard ADC (ADC_st_) maps and multi-high *b*-value ADCs (ADC_mh_) were calculated, respectively. Aquaporin 4 expression was quantified using Western blot. Relative values of ADC_st_ and ADC_mh_, AQP4 expression were compared between the sham group and the ischemia group. Correlations between ADC values and AQP4 expression were evaluated.

**Results:**

At 0.5 h after suture insertion, the value of ADC_mh_ on the lesion was obviously decreased, and there was no difference in lesion volume when compared with ADC_st_. After reperfusion, besides similar regions where ADC_st_ values decreased, we also found additional large values on ADC_mh_ within the cortex of the ipsilateral side or surrounding the lesion. The lesion evolution of the large value on ADC_mh_ was quite different from other indicators. But the total ADC_mh_ values were still significantly associated with ADC_st_. The AQP4 protein expression level was appreciably increased after middle cerebral artery occlusion (MCAO), but there was no correlation between AQP4 expression either with ADC_mh_ or ADC_st_.

**Conclusion:**

We found the large values on ADC_mh_ during the progression of cerebral infarction is varied, but there was no correlation between ADC_mh_ values and AQP4 expression. ADC_mh_ may indicate the heterogeneity of ischemia lesions, but the underlying pathological basis should be further explored.

## Highlights

– Apparent diffusion coefficient (ADC) calculated using multi-high *b*-values may assist in the individualized evaluation of cerebral ischemia pathology.
– No direct correlation was found between ADC derived from multi-high *b*-values and Aquaporin 4 (AQP4) protein expression.

## Introduction

Stroke is a significant cause of human mortality and morbidity worldwide (Benjamin et al., 2017). Ischemic stroke, which results from occlusion of a major cerebral artery, is the most common type that accounts for about 87% of all strokes (Benjamin et al., 2017; Kuts et al., 2019). The pathophysiology of ischemic stroke integrates several complex processes, among which cerebral edema is the most significant one. Since brain volume is typically limited by the rigidity of the skull, when edema occurs, even minute, it can be particularly devastating, leading to an increase in intracranial pressure and compression of brain tissue (Vella et al., 2015). As a passive osmotic-driven bidirectional water gateway (Pirici et al., 2017), Aquaporin 4 (AQP4), the most abundant water-permeable channel found in the brain (Bi et al., 2017), plays a dual role in cytotoxic and vasogenic edema (Filippidis et al., 2016; Tang and Yang, 2016; Verkman et al., 2017; Huang et al., 2019). In addition to serving as a water flux route (Tang and Yang, 2016), AQP4 is also involved in many critical events such as cell adhesion (Hiroaki et al., 2006), glial cells’ migration, glial scar formation (Saadoun et al., 2005), neuroinflammation (Zhao et al., 2018), and Ca2+ signaling pathway (Thrane et al., 2011). AQP4 is therefore known as a promising therapeutic target in cerebral edema (Tang and Yang, 2016).

Hence, it will be of great clinical significance if real-time monitoring of AQP4 expression is available. As early as 2009, Thomas Tourdias’s research has shown a correlation between apparent diffusion coefficient (ADC) values and AQP4 expression in interstitial edema (Tourdias et al., 2009). Aquaporins have even been established as a genetically encoded reporter in tumors for normal diffusion-weighted MRI (Mukherjee et al., 2016). Nevertheless, to date, there are still only a few comparative studies available on this issue (Wang et al., 2012; Schob et al., 2017), and it is a great challenge to isolate the signal that represents the permeability of AQPs in the cell membrane. Recently, a tri-component model was used to assess brain damage in Parkinson’s disease patients (Xueying et al., 2015). In this model, a bi-exponential equation using *b*-values less than 2,000 s/mm^2^ was used to calculate pure diffusion coefficients (D) and pseudo diffusion coefficients (D*) values, which represent pure water diffusion and blood perfusion, respectively, while an extra mono-exponential equation using ultra-high *b*-values (2,000–5,000 s/mm^2^) was used to quantify ultra-high ADCs that was assumed to represent the permeability of AQPs. The final result showed significantly lower in all ROIS in PD patients, which strongly suggested the relationship between multi-high ADCs values and the function of AQPs, although there was no direct evidence.

Therefore, in the current study, we hypothesized that multi-high *b*-value ADCs (ADC_mh_) might have a particular value for ischemic stroke evaluation and that the change of ADC_mh_ might be potentially related to AQP4 expression. Using the model mentioned above, we compared ADC_mh_ values in a cerebral ischemia-reperfusion rat model with a sham. We also assessed the correlation between ADC_mh_ value and AQP4 expression.

## Materials and methods

### Animal preparation

This study was approved by the institutional review board at Ningde Municipal Hospital of Ningde Normal University, and all animals received humane care in compliance with the National Institutes of Health Guide for the Care and Use of Laboratory Animals (NIH publications number 80–23, revised in 1996). Male adult Sprague–Dawley rats (250–280 g) were provided by the experimental animal center of Fujian Medical University and were housed in rooms with a 12:12 h light-dark cycle and controlled temperature at 21–25°C, with a humidity level between 50 and 60%. Rats were provided with normal food and water until 1 day before surgery. Totally 38 rats (250–280 g) were randomly divided into two groups: (1) sham group 1 (*n* = 6), (2) ischemia group (successful model, *n* = 19).

### Surgical procedure

Focal cerebral ischemia-reperfusion was induced using a middle cerebral artery occlusion (MCAO) model. Anesthesia was induced with 5% isoflurane and maintained with 2–3% isoflurane during the entire surgery. Core body temperature was monitored and maintained at 37°C by using a rectal probe and a heating pad. For the induction of ischemia, a midline neck incision was made to expose the right side common carotid artery (CCA) and the external carotid artery (ECA), both of which were surrounded by a suture. Firstly, the CCA was occluded with an aneurysm clip; then, the distal ECA was ligatured and cut off. Next, a 2% heparin pre-soaked, 4–0 monofilament nylon suture, whose tip had a silicone rubber coated (0.26 mm diameter, 4–5 mm long) was inserted into the ECA and gently advanced along the inner carotid artery until it reached the proximal anterior cerebral artery (approximately 10 mm past the carotid canal) which insured blockage of the origin of the MCA. The aneurysm clip occluded CCA was then removed immediately. The monofilament nylon suture was extracted 1 h later. A Laser Doppler Flowmetry (PERIMED AB, PeriFlux System 5000, Stockholm, Sweden) was used to monitor local cerebral blood perfusion, and a minimum initial reduction of 75% in the laser Doppler reading after MCA occlusion was considered a successful model. The Sham group suffered the same surgical procedure without MCA occlusion.

MRI examination was conducted with a 3.0-T MR scanner (General Electric, Milwaukee, WI, USA) using a dedicated four-channel phased array rat head coil assembly (WK602, Magtron Inc., Zhejiang, China). Rats were anesthetized with 2–3% isoflurane throughout the MRI examination. Rats were placed supinely into a plastic holder. Consecutive MRI examination was executed at five time points: 30 min after suture insertion (0.5 h_co_) and 1, 3, 6, and 24 h after reperfusion (1, 3, 6, and 24 h_re_). The imaging protocol included T2 FLAIR (TR = 8,450 ms, TE = 145 ms, FOV = 50 mm, image matrix = 256 × 256, echo train length = 32, slice thickness = 2 mm, slice interval = 0 mm, NEX = 1, Flip angle = 111°, bandwidth = 62.5 kHZ), Diffusion-weighted images (TR = 3,000 ms, TE = 83.7 ms, FOV = 50 mm, image matrix = 128 × 128, slice thickness = 2 mm, slice interval = 0 mm, and width = 166.7 kHZ). Diffusion-weighted imaging (DWI) sequences was performed with standard *b*-values (0, 1,000 s/mm^2^) and multi-*b*-values including 19 different *b*-values (0, 30, 50, 80, 100, 150, 200, 300, 500, 800, 1,000, 1,300, 1,700, 2,000, 2,500, 3,000, 3,500, 4,000, and 4,500 s/mm^2^). The number of excitations (NEX) for *b* = 0–4,500 s/mm^2^ were 1, 1, 1, 1, 1, 1, 1, 1, 2, 2, 2, 2, 2, 2, 4, 4, 6, and 8, respectively. The diffusion-time of each *b* value is about 49.5 ms. The multi-*b*-value DWI scan time was about 6 min. The whole MRI examination approximately lasted for 10 min for each animal. The quantitative standard ADC (ADC_st_) and ADC_mh_ maps were obtained according to the method described by Xueying et al. (2015). Briefly, ADC_st_ map was calculated from the standard DWI sequence using a mono-exponential model as follows:


$$\frac{\text{S}}{{\text{S}}_{0}}=e\,x\,p\;(-\text{b}\cdot {\text{ADC}}_{s\,t}),$$

Where *S* is the diffusion-weighted signal intensity for the *b*-value, and *S*0 is the signal intensity obtained with the *b*0-value. ADC_mh_ maps were calculated as follows:


$$\frac{\text{S}}{{\text{S}}_{0}}=\text{e}\,x\,\text{p}\;(-\text{b}\cdot {\text{ADC}}_{\text{uh}}),\text{b}\geq 2000\,\frac{\text{sec}}{{\text{mm}}^{2}}$$

In this study, we only focus on the comparison of ADC_st_ and ADC_mh_. Thus according to previous research (Xueying et al., 2015), the same mono-exponential equation is used to quantify the ADC_mh_ by fitting the six multi-high *b*-values (2,000, 2,500, 3,000, 3,500, 4,000, and 4,500 s/mm^2^). All algorithms were calculated on the workstation (General Electric Advantage Workstation 4.6), which allowed the extraction of ADCst, ADC_mh_ maps on a pixel-by-pixel basis (Xueying et al., 2015).

Regions of interest (ROIs) were determined by two independent neuroradiologists with 8 and 10 years of clinical experience, respectively. To minimize the impact of individual differences, we calculated lesion volumes only in rats who underwent complete consecutive MRI examinations, and the final result was shown as the percentage of the final infarction area of the individual. The specific calculation formula of relative lesion volume is as follows:


$$r\,V_{n}=100\times \left(\frac{S_{i\,n}}{S_{a\,n}}÷\frac{S_{i\,24}}{S_{a\,24}}\right)$$

**FIGURE 1:**
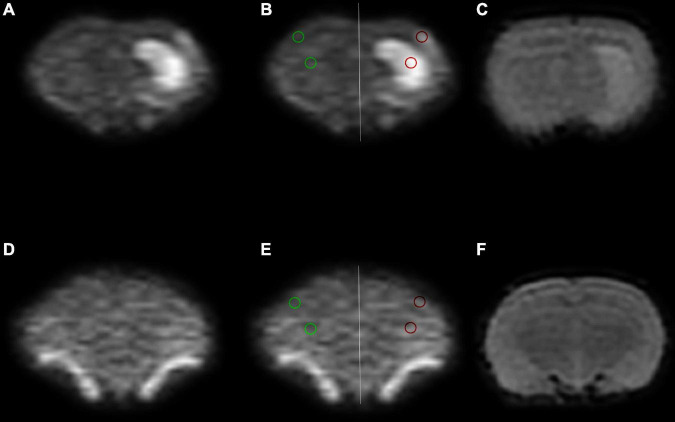
Region of interest (ROI) placement diagram. **(A–C)** The ischemia group. **(D–F)** The sham group. The selected ROIs are shown on the DWI_4000_ maps. ROI1 (red circle) was bounded by the lesions on DWI4000, and ROI2 (green circle) was bounded by cortical regions, and the same sized ROIs were drawn in the symmetrical region of the bilateral lobes, avoiding the interference areas, such as the cerebrospinal fluid and blood vessels.

Where *n* is the time point, *S*_*i*_ is the sum of the lesion area of each layer, and *S*_*a*_ is the sum of the whole brain area of each layer. ADC values were measured in two ROIs. ROI1 was bounded by the lesions on DWI_4000_, and ROI2 was bounded by cortical regions but avoided the folding artifacts (Figure 1). The specific calculation formula of relative ADC (rADC) is as follows:


$$r\,A\,D\,C=100\times \left(A\,D\,C_{i}-A\,D\,C_{c}\right)÷A\,D\,C_{c}$$

Where ADC_i_ is the ADC of ROI on the ipsilateral side, and ADC_*c*_ is the ADC of mirror-symmetric ROI on the contralateral side.

### TTC staining

Rats were anesthetized with chloral hydras (10%) intraperitoneally (300 mg/kg) 24 h after MACO, perfused intracardially with 100 ml of cold 10 mM sodium phosphate-buffered saline (PBS; pH 7.4), and then decapitated quickly. Brains were then rapidly removed and sectioned coronally at 2 mm intervals. The brain slices were following incubated in 2% 2,3,7-triphenyl tetrazolium chloride (TTC) solution for 30 min at room temperature.

### Western blot

Aquaporin 4 protein expression level was detected by Western blot. After MRI examination, rats were rapidly perfused intracardially with cold PBS and then decapitated quickly. The Infarcted cerebral hemisphere was isolated on ice and homogenized. The concentration of protein in the resulting supernatants was calculated using a BCA protein assay kit. Samples containing approximately 40 μg of protein were loaded, separated using 10% SDS-polyacrylamide gel electrophoresis, and then transferred onto 0.45 μm polyvinylidene difluoride (PVDF) membranes (Bio-Rad, CA, USA). After blocking with Tris–buffered saline containing 5% skimmed milk for 2 h, the blot was then incubated overnight at 4°C with primary antibodies, including AQP4 (1:1300, Santa Cruz Biotechnology, TX, USA, SC-20812), β-actin (1:1000, Beyotime, Shanghai, China, AA128). The blot was subsequently incubated with horse radish peroxidase-labeled antibody (1:1000, Beyotime, Shanghai, China, A0208) for 1 h at RT. The membrane was then incubated in ECL solution, and the specific bands were captured and quantified with a ChemiDocMP (Bio-Rad, CA, USA). The AQP4 protein levels were normalized to β-actin.

### Immunohistochemistry

Rats were *trans*-cardiac perfused with ice-cold PBS and 4% PFA solution. Brains were post-fixed in 4% PFA solution at 4°C overnight and then cryoprotected in 20% sucrose overnight. Cryostat sections (40 μm) were stained with antibodies against AQP4 (1:100; Santa Cruz Biotechnology, TX, USA, SC-20812), and GFAP (1:100; Santa Cruz Biotechnology, TX, USA, SC-56395) which label the astrocyte cells. Double staining of GFAP and AQP4, were performed consecutively with their primary and secondary antibodies, respectively. Fluorescent stained sections were analyzed by confocal microscopy.

### Statistical analysis

Statistical analysis was performed using SPSS 22.0 for Windows. Data are presented as the mean ± SD. One-way analysis of variance (ANOVA) followed by *post hoc* Fisher’s LSD and Tamhane’s T2 tests were used for within-group comparisons. Unpaired *t*-tests were applied for between-group comparisons of the relative lesion volumes, rADC, and protein level. Spearman correlation analysis was performed to assess the correlation between AQP4 expression and ADC parameters, or between ADC_mh_ and ADCst. *P* < 0.05 was considered to indicate statistical significance (Figure 1).

## Results

### Animals

**FIGURE 2:**
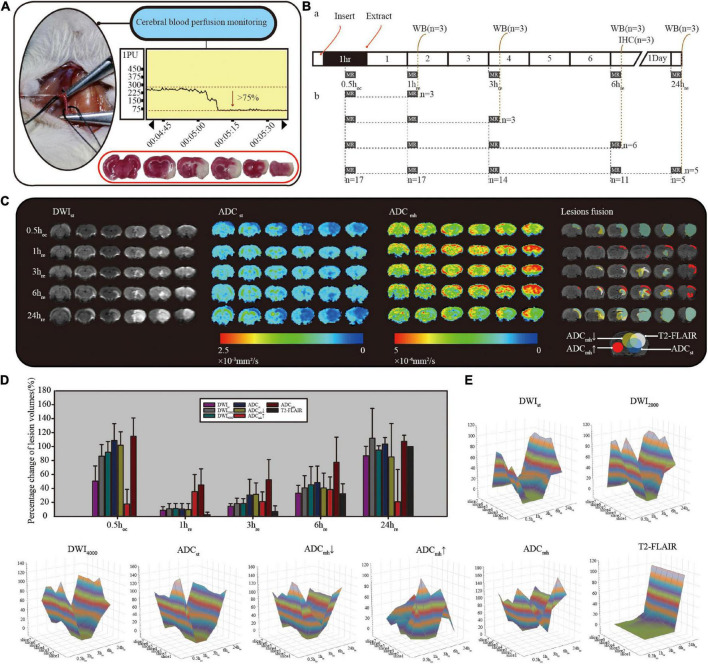
**(A)** Criteria for successful model: a minimum initial reduction of 75% in the laser Doppler reading. 2,3,7-triphenyl tetrazolium chloride (TTC) staining is inside the red border, with normal tissue stain in red and infarction stain in white. **(B)** Diagram of MRI data collection and sampling of rats. **(C)** Comparison diagram of different sequences in single rat with consecutive MRI detection. ADC_mh_↓ represents the small value on the ADC_mh_ map, ADC_mh_↑ represents the large value, and ADC_mh_ represents all lesions with the abnormal signal. **(D)** Histogram of the normalized relative lesion volumes. **(E)** The dynamic evolution of relative lesion volumes at seven slices. All data were normalized by their respective infarction volume at 24 h on T2-FLAIR. All of the diffusion-weighted imaging (DWI) at different *b*-values, ADC_st_ and ADC_mh_↓ had similar lesion evolution, while ADC_mh_↑ was quite different.

Overall, 38 rats were sacrificed in this experiment, 6 rats for sham control and 32 rats for ischemia model induction. With the laser flow meter monitoring, only 19 stroke models were successfully established, of these, 17 rats received MRI detection before being sacrificed (Figure 2B) and the remaining 2 were sacrificed for TTC staining directly 24 h after ischemia. There was obvious infarction shown in TTC staining (Figure 2A with red border).

### MRI results

To observe the dynamic evolution, we collected MRI data at five-time points: 30 min after suture insertion (0.5 h_co_) and 1, 3, 6, and 24 h after reperfusion (1, 3, 6, and 24 h_re_). As shown in Figure 2C, lesions appear as early as 0.5 h after suture insertion on standard DWI with mildly increased signal intensity, and similar range lesions with obviously reduced ADC values are found both on ADC_st_ and ADC_mh_. After reperfusion, the infarct size that with typical DWI hyperintensity and limited ADC dispersion grew with time. Whereas on ADC_mh_ maps, besides similar regions where ADC_st_ values decreased, we also found additional large values on ADC_mh_ within the cortex of the ipsilateral side or surrounding the lesion. The percent change of relative lesion volumes on each sequence at different timepoint are shown in Figure 2D and Table 1. To minimize the impact of individual differences, infarct volumes were calculated in only five rats who underwent complete consecutive MRI scans. In addition, the final infarct volume of T2-FLAIR at 24 h_re_ of individuals was used as the denominator to normalize the infarct volume within different sequences. At 0.5 h after suture insertion, the lesion volumes on DWI and ADC were close to the final infarct volume, both at conventional *b*-value and multi-high *b*-values (*p* > 0.05). At every time point, the volume of the large value on ADC_mh_ maps was very close to that of ADC_st_ (*p* > 0.05). And the region of the large value on ADC_mh_ arose at all time points. All indicators except ADC_mh_↑ showed an increasing change pattern of lesion volume after MACO. The dynamic evolution of relative lesion volumes at seven slices is presented in Figure 2E.

**TABLE 1:** Percent change of relative lesion volumes on each sequence at different time points (%).

Relative lesion volumes (%)	0.5 h_co_	1 h_re_	3 h_re_	6 h_re_	24 h_re_
DWI_st_	50.41 ± 21.79	8.60 ± 5.32***	14.35 ± 3.81***	33.15 ± 11.31***	86.78 ± 13.58
DWI_2000_	86.30 ± 16.58	10.82 ± 7.38***	18.23 ± 7.85***	40.78 ± 17.46***	92.25 ± 5.88
DWI_4000_	91.71 ± 15.57	11.22 ± 7.11***	18.30 ± 7.02***	45.15 ± 26.11***	94.88 ± 6.34
ADC_st_	108.80 ± 23.94	10.49 ± 7.87**	30.42 ± 22.68***	48.33 ± 23.80***	103.68 ± 9.30
ADC_mh_↓	101.82 ± 19.29	9.66 ± 8.58**	31.41 ± 16.40***	40.58 ± 21.35***	85.16 ± 47.87
ADC_mh_↑	17.66 ± 20.96*	35.33 ± 24.55	21.04 ± 13.98***	38.36 ± 18.12	20.74 ± 45.18
ADC_mh_	114.74 ± 26.27	44.99 ± 23.06	52.40 ± 29.00***^###^	77.42 ± 36.06***^##^	107.48 ± 8.80
T2-FLAIR	0.00 ± 0.00***	2.36 ± 3.47***	6.93 ± 8.19***	32.33 ± 14.24***	100.00 ± 0.00

*p < 0.05, vs. T2-FLAIR at 24 h_re_. **p < 0.01, vs. T2-FLAIR at 24 h_re_. ***p < 0.001, vs. T2-FLAIR at 24 h_re_. ^##^*p* < 0.01, vs. ADC_st_. ^###^*p* < 0.001, vs. ADC_st_.

We measured ADC values in two ROI types, type 1 was bounded by the lesions on DWI4000, and then another one was bounded by cortical regions, we also measured ADC values in the symmetric mirror ROIs of the contralateral side. The relative ADC was then calculated. Mean rADC values of standard and multi-high *b*-values at different time points are summarized in Table 2. Compared with the sham group, both the rADC_st_ and rADC_mh_ of the lesion in the ischemia group were statistically different at each time points. However, at each time point, the ADC_mh_ value was higher than that of ADC_st_, and there were statistical differences between them at 0.5 h_co_ and 3 h_re_ time points (*p* < 0.05). In the cortex, the small value were detected on both ADC_st_ and ADC_mh_ maps at 0.5 h_co_ and 24 h_re_ time points, which were much lower than the sham group (*p* < 0.001). From 1 to 6 h after MACO, we detected an increased ADC_mh_ values in cortical regions, the relative change was much higher than that of ADC_st_ (*p* < 0.01). Whereas compared to the sham group, there were statistically different only at 6 h_re_ time point.

**TABLE 2:** Mean relative ADC (rADC) values at different time points (%).

	Lesion		Cortex	
	rADC_st_ (%)	rADC_mh_ (%)	rADC_st_ (%)	rADC_mh_ (%)
Sham	0.27 ± 0.96	−0.62 ± 0.75	−0.33 ± 0.83	0.22 ± 1.37
0.5 h_co_	−17.20 ± 6.30***	−12.21 ± 4.96***^#^	−9.63 ± 7.55***	−5.64 ± 6.69**
1 h_re_	−9.30 ± 7.00**	−6.64 ± 6.52*	−0.29 ± 3.9	4.21 ± 3.14^##^
3 h_re_	−13.21 ± 5.86***	−8.14 ± 6.02*^#^	−0.85 ± 2.97	2.68 ± 3.30^##^
6 h_re_	−21.38 ± 7.42***	−16.13 ± 7.46***	−2.56 ± 2.93	6.13 ± 4.23*^###^
24 h_re_	−43.57 ± 8.21***	−37.54 ± 6.66***	−33.98 ± 6.33***	−29.30 ± 4.35**

*p < 0.05, vs. sham group. **p < 0.01, vs. sham group. ***p < 0.001, vs. sham group. ^#^*p* < 0.05, vs. ADC_st_. ^##^*p* < 0.01, vs. ADC_st_. ^###^*p* < 0.001, vs. ADC_st_.

### Correlation between AQP4 expression and diffusion parameters

**FIGURE 3:**
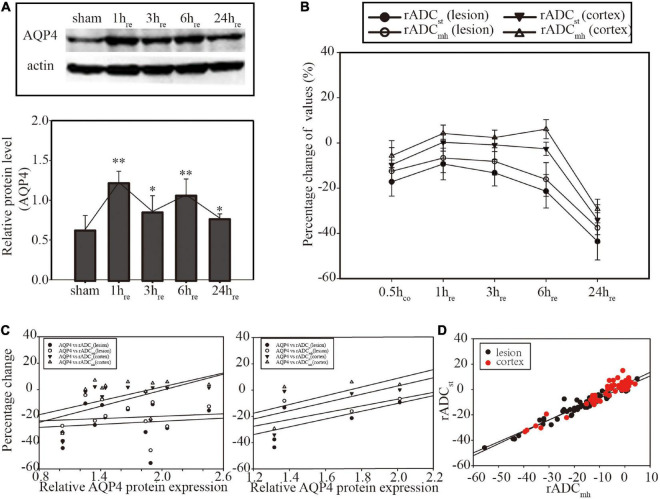
**(A)** Western blot of Aquaporin 4 (AQP4) expression and histogram for relative quantitative analysis. **(B)** Linear graph of the dynamic evolution of rADCs. **(C)** Correlation analysis between AQP4 protein expression and rADCs at the individual level (former, *n* = 12) and the group level (latter, *n* = 4). No significant correlation was found (*p* > 0.05). **(D)** Correlation analysis between rADC_st_ and rADC_mh_. Significant correlation was found in both focal (*p* < 0.01) and cortical areas (*p* < 0.05). *p < 0.05, vs. sham group. ^**^p < 0.01, vs. sham group.

Western blot result of AQP4 expression is shown in Figure 3A. Compared with the sham group, the ischemia group showed an increased level of AQP4 that peaked at 1 and 6 h_re_. Results of Spearman correlation analysis between AQP4 level and each ADC parameter in lesion and cortex are shown in Figure 3C. However, there was no significant correlation between AQP4 protein expression and any kind of ADC. In the ischemia group, there was a significant correlation between rADCst and rADC_mh_ in both ROIs (*p* < 0.01 in the lesion and *p* < 0.05 in the cortex) (Figures 3B,D).

**FIGURE 4:**
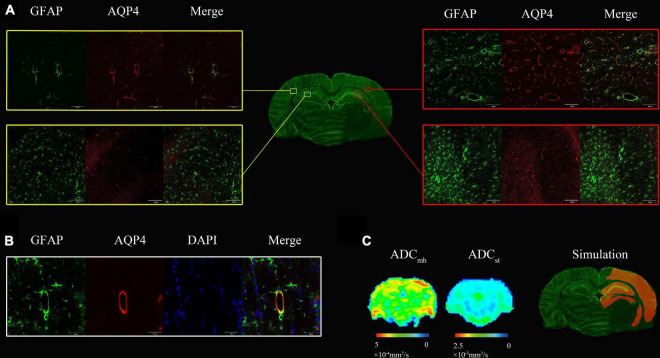
**(A)** At 6 h after MACO, there was an extensive increase level of both Aquaporin 4 (AQP4) (red) and GFAP (green) in the ipsilateral cerebral cortex and hippocampus compared with the contralateral side. **(B)** The AQP4 colocalized with the GFAP staining surrounding blood vessels. **(C)** There were localized increased multi-high *b*-value ADCs (ADC_mh_) value in the ipsilateral cerebral cortex, but no visible change on the ADC map. The last one is the simulation diagram of regions with increased AQP4 expression that is highlighted in orange, which was much more extensive than the hyperintensity of ADC_mh_.

Results of confocal immunofluorescence are shown in Figure 4. There was an extensive increase level of AQP4 in the ipsilateral cerebral cortex and hippocampus (Figure 4A) at 6 h_re_. We also found increased AQP4 expression in the border of the lesion and the area of white matter adjacent to the lesion (not shown). The final diagram of Figure 4C simulates the region of increased AQP4 expression. Most of the increased AQP4 was distributed in astrocyte end feet that surrounded blood vessels. At the level corresponding to the brain biopsy, there were localized increased ADC_mh_ value in the ipsilateral cerebral cortex, but there was no visible change on the corresponding ADC map (Figure 4C).

## Discussion

In this study, we found that in addition to predicting lesions like ADC_st_, ADC_mh_ which is generated by six multi-high-*b* values (2,000–4,500 s/mm^2^) may also indicate individual heterogeneity. As early as 0.5 h after suture insertion, the value of ADC_mh_ on the lesion was obviously decreased, and there was no difference in lesion volume when compared with ADC_st_ (Table 1). Although the relative change value of ADC_mh_ was higher than that of ADC_st_, it was still significantly lower than the sham control (Table 2). These results suggest that ADC_mh_ can predict infarction as well as ADC_st_ before reperfusion. The assumption that ADC_mh_ can indicate individual heterogeneity is based on the large value with a large degree of dispersion occurring on ADC_mh_ maps. Before reperfusion, the large value on ADC_mh_ was only observed in two out of five rats that underwent consecutive MRI examination, whereas, after reperfusion, it was observed in all rats within 6 h. The volumes and values of these regions of the large value on ADC_mh_ is varied greatly in different rats, thus resulting in a high degree of standard deviation. Their common feature is that they are mostly distributed in the ipsilateral cerebral cortex or around the lesions.

Multi-high *b*-value ADCs from multi-high-*b* values has been proved to be a useful parameter for evaluating brain damage in Parkinson’s disease (Xueying et al., 2015), glioma grading (Hu et al., 2017; Tan et al., 2018), early detection of kidney damage in diabetic nephropathy (Wang et al., 2017), and monitoring the pathophysiological changes of AD (Xue et al., 2019). In our study, multi-high *b*-value ADC was applied to a focal cerebral ischemia rat model. However, the pathological basis of the large value on ADC_mh_ we found is unknown. Intra-voxel incoherent motion (IVIM) has been proved to distinguish pure water diffusion from pseudodiffusion or blood perfusion. Blood perfusion signals have greater influence on DWI images when *b*-value is less than 200 s/mm^2^, while true water diffusion with a slower flow mainly contributes to the signal acquired with *b*-values larger than 200 s/mm^2^ (Xueying et al., 2015; Paschoal et al., 2018; Szubert-Franczak et al., 2020). When the *b*-value is greater than 2,000 s/mm^2^, it is most likely to reflect the effect of Aquaporin-4 on water transport (Zeng et al., 2018). According to previous studies (Xueying et al., 2015; Hu et al., 2017; Wang et al., 2017; Xue et al., 2019), ADC_mh_ may be associated with water transportation by aquaporins. We, therefore, speculated whether the large value on ADC_mh_ represents the high expression of AQP4 which plays a crucial role in cerebral ischemia. AQP4 expression was significantly upregulated after MACO in this study, which is by previous studies (Murata et al., 2020; Vandebroek and Yasui, 2020; Wang et al., 2020a,b; Shi et al., 2021). In this study, AQP4 expression peaked at 1 h after MACO, decreased slightly at 3 h, recovered to a sub-peak by 6 h, and then decreased at 24 h, but remained above the base level. These highly expressed AQP4 proteins were widely distributed in the ipsilateral cerebral cortex, the broader of lesions, and the hippocampus as well. And AQP4 was mainly expressed on the astrocyte endfeet around vessels. These findings are consistent with a previous study executed in a mouse model of cerebral ischemia (de Castro Ribeiro et al., 2006). Whereas on the ADC_mh_ maps, the distribution of the large value was very limited which was significantly smaller than the expression of AQP4 protein in the same section (Figure 4C). We further analyzed the correlation between the level of AQP4 protein isolated from ipsilateral brain homogenate and the ADC_mh_ values in the focal lesions and ipsilateral cortex, respectively, but no significant correlation was found either at the individual level or at the group level. These results indicate that ADC_mh_ does not represent AQP4 protein expression directly in our model.

In some brain tumor (Mukherjee et al., 2016; Tan et al., 2018), and hydrocephalus (Tourdias et al., 2009) models, AQP4 has been proved to be correlated with single ADC values or multi-parameter ADC values, but such a rule cannot be verified in our experimental model. However, it is not enough to directly deny the correlation between ADC from multi-high *b*-values and AQPs. The tri-exponential models, from which the ADC_mh_ derived in this study, are thought to be an over-fitting model (Zeng et al., 2018). Obata et al. (2018) recently showed that the water exchange time of non-AQP4-expressing cells is longer than that of AQP4-expressing cells by multi-*b* and multi-diffusion-time diffusion-weighted MRI and coherent anti-Stokes Raman scattering imaging. And Urushihata et al. (2021) found that the water exchange time in Aquaporin-4 knockout mice was approximately 2.5 times longer than that in wild-type mice. Cell membrane water permeability is regulated by AQP4, and the effect of cell membrane water permeability is on DWI measurement is affected by the diffusion time (Obata et al., 2018; Urushihata et al., 2021). Therefore, appropriate diffusion time should be optimized to reduce the effect of non-AQP4-expressing cells on signal attenuation. Additionally, the edema mechanism of cerebral ischemia is much more complex than the relatively simple edema pattern of tumors and hydronema. Our results show that increased AQP4 is accompanied by increased astrocytes and microvessels (Figure 4). The role of these complex microenvironment changes in the pathogenesis of cerebral ischemia has not been fully elucidated, and the dual role of AQP4 relies on the proportion of subtypes and the change of molecular structure rather than the amount (Barati Farimani et al., 2014; Abe et al., 2017; Lisjak et al., 2017; Palazzo et al., 2019; Simone et al., 2019). Sun et al. (2022) recently showed that peri-infarct AQP4 polarization correlated positively with the ultra-high *b*-values of ADC in rat at 14 days after stroke. This may indicate that the large value on ADC_mh_ may reflect a change in AQP4 functionality, rather than a change in quantity. The time of the large value on ADC_mh_ appearance between five rats with continuous MRI scanning in the ischemia group was different, which may indicate the heterogeneity of lesions among different individuals. AQP4 plays a dual role in the formation and regression of vasogenic and cytotoxic edema (Salman et al., 2022). Whether the changes of ADC_mh_ in different periods have an impact on the prognosis of the disease needs further exploration to elucidate the mechanism behind it, so as to help the individual evaluation of the disease.

There are still several limitations in this study. First, the values of slow and fast ADC that can also be derived from the tri-exponential models were not evaluated in our study, the main focus of this study was on the comparison between ADC_mh_ and ADC_st_. Second, as mentioned above, we only compared the AQP4 protein expression level and distribution with ADC_mh_ values. The functional changes of AQP4 and another possible pathological basis should be further explored. Third, the relationship between ADC_mh_ and other members of Aquaporins including AQP1 and AQP9 was not evaluated in our research and needs further exploration. Finally, the ischemic cascade is a dynamic evolutionary process. During a longer period of scanning, the disease is constantly evolving and progressing, which may affect the result.

In conclusion, this is an original study using ADC_mh_ to evaluate cerebral ischemia, and the large value on ADC_mh_ which is found during the progression of cerebral infarction is varied, which may indicate the heterogeneity of lesions and thus provide assistance for individualized evaluation. The current results have not confirmed the correlation between ADC_mh_ values and AQP4 expression, but the underlying pathological basis of the large value on ADC_mh_ is still worth further exploration.

## Data availability statement

The raw data supporting the conclusions of this article will be made available by the authors, without undue reservation.

## Ethics statement

This animal study was reviewed and approved by the Institutional Review Board at Ningde Municipal Hospital of Ningde Normal University.

## Author contributions

LS contributed to analysis and manuscript. BY and QC performed the experiment. QC performed the data analyses and wrote the manuscript. TZ and PX helped to perform the analysis with constructive discussions. DW contributed to the conception of the study. All authors contributed to the article and approved the submitted version.
